# Extended reality for perforator visualization in deep inferior epigastric perforator autologous breast reconstruction: A systematic review

**DOI:** 10.1016/j.jpra.2025.11.025

**Published:** 2025-11-24

**Authors:** Killian Zijlstra, Chien Nguyen, Koen Willemsen, Henk Coert, Eveline Corten, Wiesje Maarse

**Affiliations:** a Division of Surgical Specialties, University Medical Center Utrecht, 3D Lab, Utrecht, The Netherlands; b Department of Plastic, Reconstructive and Hand Surgery, University Medical Center Utrecht, Utrecht, The Netherlands; c Department of Orthopaedics, University Medical Center Utrecht, Utrecht, The Netherlands; d Department of Plastic, Reconstructive and Hand Surgery, Erasmus MC Cancer Institute, University Medical Center Rotterdam, Rotterdam, The Netherlands

**Keywords:** Autologous breast reconstruction, 3D medical technology, Virtual reality, Augmented reality, Extended reality

## Abstract

**Background:**

Extended Reality (XR) technology is rapidly advancing and has shown promise in improving perioperative outcomes across various surgical specialties.

**Objective:**

This systematic review aimed to evaluate the use of XR for perforator vessel visualization in autologous breast reconstruction.

**Method:**

A systematic search was conducted following PRISMA guidelines, consulting Embase, Medline (Ovid), Web-of-Science, Cochrane, and Google Scholar on June 23, 2025. Articles describing the use of XR for perioperative perforator visualization in free flap breast reconstruction were included. Outcome measures included perforator identification rate, virtual model construction time, preoperative planning duration, flap dissection time, usability, complications, and costs.

**Results:**

Ten articles were included, all focused on XR in deep inferior epigastric perforator (DIEP) flap breast reconstruction. Three XR modalities were identified: virtual reality (VR), augmented reality (AR) projection, and AR glasses. Perforator identification using XR ranged from 61.7 % to 100 %, with AR outperforming handheld Doppler ultrasound (US) in several studies. XR use decreased operative time, with AR reducing intraoperative perforator localization time from 20 min using handheld Doppler US to 2.3 min. The use of XR did not result in significant additional costs, and no differences in complication rates were identified.

**Conclusion:**

XR may assist surgeons in perioperative perforator visualization during DIEP flap breast reconstruction by enhancing anatomical understanding. However, current evidence is constrained by small, low-quality studies and comparisons with handheld Doppler rather than the gold standard computed tomography angiography (CTA). Whether XR offers clinically meaningful advantages over conventional CTA imaging remains uncertain, as this was not explored in the included articles. Larger, high-quality comparative studies are needed to establish its true clinical value.

## Introduction

Breast cancer is the most frequently diagnosed cancer among women worldwide.^1^ Breast cancer treatment often requires a multimodal approach, which may include (neo)adjuvant systemic therapy, surgery, and radiotherapy. Post-mastectomy breast reconstruction has become an essential part of treatment, as it significantly improves health-related quality of life, body image, and sexual well-being, especially when performed in the same surgery as the mastectomy, known as immediate, single stage, or one step reconstruction.^2^, ^3^, ^4^

Breast reconstruction (BR) is categorized into implant-based breast reconstruction (IBR) and autologous breast reconstruction (ABR), including free flaps, pedicled flaps, and lipofilling. Among these, free flap reconstruction is the most common type of ABR worldwide. While IBR is generally quicker and less complex, ABR tends to yield higher patient satisfaction.^5^

Numerous donor site regions have been described for free flap ABR including skin, fat, and muscle tissues.^6^, ^7^, ^8^, ^9^, ^10^ Abdominal-based free tissue flaps, such as the deep inferior epigastric perforator (DIEP) flap, are considered as favorable, due to its natural appearance, tissue quality of the abdomen, and donor site surplus.^11^^,^^12^

Abdominal wall vasculature is known to have a high variety of branching patterns as first described by Moon and Tayler in 1988.^13^^,^^14^ Operative success is related to the perioperative identification of the most suitable and dominant perforators in terms of laterality, caliber, and intramuscular course.^15^ Computed tomography angiography (CTA) is considered the gold standard for preoperative imaging of perforators, facilitating vessel selection during preoperative planning.^16^, ^17^, ^18^ Other options for perforator selection are magnetic resonance imaging or angiography (MRI, MR-A) and handheld Doppler ultrasound (US), which are often used for preoperative perforator visualization.^19^^,^^20^

However, these imaging modalities display two-dimensional (2D) images in different planar views on a monitor and provide no three-dimensional (3D) visualization of the perforators and other relevant structures. Consequently, reconstructive surgeons must accurately interpret and mentally reconstruct 2D images into 3D anatomy, while simultaneously navigating a 3D surgical environment during vessel dissection. A promising advancement for more 3D insight in both preoperative planning and intraoperative navigation for autologous free flap BR is the integration of extended reality (XR). XR facilitates the 3D visualization of conventional 2D images that can aid the surgeon in the field of reconstructive surgery.^21^, ^22^, ^23^

XR is an umbrella term that encompasses a spectrum of technologies enabling users to immerse and interact with 3D computer-simulated environments, ranging from complete immersion in a virtual environment to subtle virtual augmentations of the physical world. Examples of XR include augmented reality (AR), virtual reality (VR), and mixed reality (MR).^24^

Research has shown that the preoperative use of XR improves the understanding of key anatomical sites during surgery, which led to an improved understanding of complex and variable vasculature, which ultimately contributes to a reduction in operative time.^25^

While recent systematic reviews have begun exploring the role of XR for perioperative perforator visualization in DIEP BR, a consistent conceptual definition of XR remains lacking.^26^, ^27^, ^28^ In contrast to prior reviews by Lacey et al. and Ozmen et al., which categorize 3D reconstructed CTA models viewed on conventional 2D monitors as XR, this systematic review adopts a more stringent definition.^26^^,^^27^ The term XR is reserved for platforms that enable stereoscopic and immersive engagement with 3D data, excluding passive 2D viewing, which lacks this depth perception.^28^ Therefore, this systematic review aims to evaluate the current applications, outcomes, and methodological quality of XR in this context, and to identify knowledge gaps for future research.

## Material/Patients and methods

The PRISMA guidelines were applied during the literature search and the writing of this systematic review.^29^

### Search strategy

On June 23, 2025, a systematic search string was conducted using several databases. No time restrictions were applied, covering articles from the earliest records available in the database to the present. These databases were Embase, Medline (Ovid), Web of Science Core Collection, Cochrane Central Register of Controlled Trials, and Google Scholar. The complete search strategies and their results per database can be found in Appendix 1.

### Article selection

All articles retrieved through the search method were screened for duplicates. Two independent authors (KZ and CN) reviewed all titles and abstracts for eligibility while remaining blinded to each other’s results. If the title and abstract did not clarify whether an article should be included, the full text was assessed. Disagreements between the two authors were resolved through discussion, with a third reviewer available if consensus could not be reached. All citations from included articles were also screened for additional relevant studies.

Articles included in this systematic review describe the perioperative visualization of perforators in the donor site using XR for autologous free flap BR. Furthermore, the article must assess one or multiple of the following outcome measures: perforator identification rate, duration of virtual model construction, duration of preoperative planning, operative time, the usability of XR as experienced by the user, complications, and costs related to XR.

The exclusion criteria for this review encompassed studies involving animals, cadavers, or studies that lacked intraoperative verification of the perforators. Additionally, studies were excluded if the full text was not available in English, if no outcome measures were reported, or if BR was not the purpose of the surgery. Finally, only peer-reviewed, published literature was included. Grey literature, such as conference abstracts, dissertations, or master's theses, was excluded.

XR was defined as an umbrella term for immersive stereoscopic technologies such as AR, VR, and MR. XR enables users to immerse themselves in and interact with computer-simulated 3D environments, meaning a three-dimensional model viewed on a regular monitor or tablet in a non-stereoscopic way was not considered to be XR.^24^^,^^28^

### Data extraction

The following data was extracted from the included articles in this systematic review: the type of free flap used for BR, the type of XR technology, the registration method, the imaging modality, the segmentation software used for construction of the 3D model, and the anatomical structures that were visualized within the XR environment.

Additionally, the following reported outcome measures were extracted: perforator identification rate, duration of virtual model construction, duration of preoperative planning, operative time, the usability of XR as experienced by the user, complications, and costs related to XR.

### Quality assessment

The Quality Assessment for Diverse Studies (QuADS) tool, designed for systematic reviews involving heterogeneous study designs, was used to evaluate all articles.^30^ Two independent authors (KZ and CN) conducted the assessments while blinded to each other’s results. Any disagreements were resolved through discussion and consensus, with a third reviewer available if consensus could not be reached.

## Results

### Literature search

**Figure 1:**
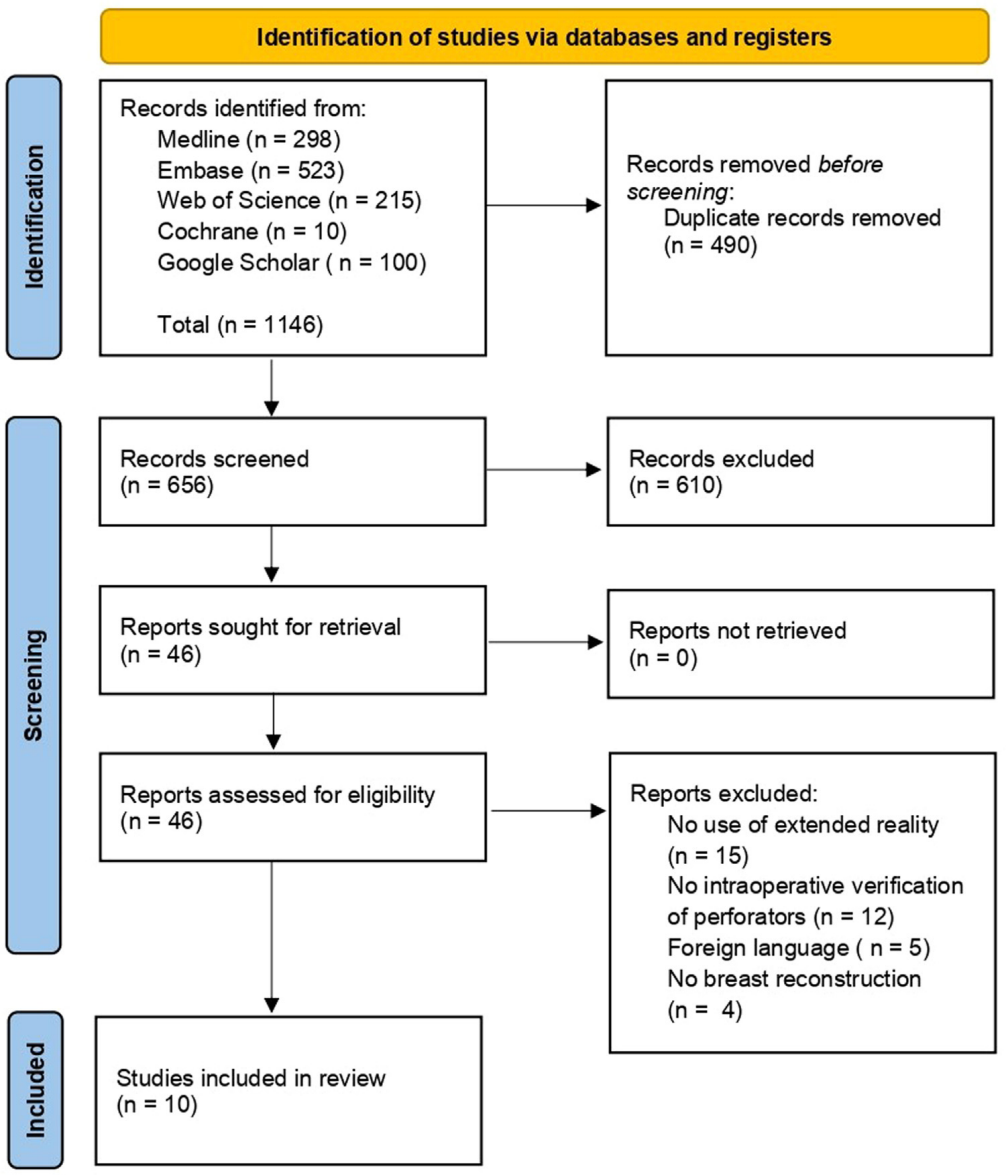
Flowchart of article selection.

The literature search yielded 1146 articles of which 490 were duplicates. Of the remaining 656 articles, 610 were excluded after review of title and abstract. 46 articles were assessed in full-text form, after which ten articles were included in this systematic review.^31^, ^32^, ^33^, ^34^, ^35^, ^36^, ^37^, ^38^, ^39^, ^40^ No eligible articles were identified through the process of citation searching or other methods. Figure 1 provides a complete overview of the article selection process.

### Study characteristics

The ten articles included in this systematic review were published between 2015 and 2024.^31^, ^32^, ^33^, ^34^, ^35^, ^36^, ^37^, ^38^, ^39^, ^40^ Among the ten articles, nine were pilot studies, and one was a randomized controlled trial (RCT). Study characteristics and the quality assessment are summarized in Table 1. The full quality assessment can be found in Appendix 2.

**Table 1:** Study characteristics.

Study	Study design	Number of patients	XR modality	Flap type	Primary outcome measurement	Quality assessment (QuADS criteria)
Hummelink et al.^31^	Pilot study	9	AR projection, PicoPix PPX2480 (Philips, Eindhoven, the Netherlands)	DIEP	Intraoperative verification of perforator using AR versus handheld Doppler US	8/39
Hummelink et al.^32^	Pilot study	6	AR projection, in-house created device	DIEP	Preoperative planned flap volume compared to intraoperative findings	10/39
Hummelink et al.^33^	RCT^a^	Study group: 33 Control group: 27 Total: 60	AR, projection, in-house created device	DIEP	Intraoperative verification of perforators using AR versus handheld Doppler US	29/39
Fitoussi et al.^34^	Pilot study	12	AR, Stereoscopic smart-glasses. (Brand not specified)	DIEP	Perforator identification with AR confirmed with handheld Doppler US and CTA	7/39
Masterton et al.^35^	Pilot study	2	AR, HoloLens 1 (Microsoft, Redmond, WA)	DIEP	Subjective feasibility	3/39
Freidin et al.^36^	Pilot study	30	VR, HTC Vive (HTC, San Francisco, Calif.)	DIEP, free TRAM	Usability of VR in addition to CTA	20/39
Seth et al., ^37^	Pilot study	5	AR, HoloLens 2 (Microsoft, Redmond, WA)	DIEP	Perforator identification with AR confirmed with handheld Doppler US	8/39
Berger et al.^38^	Pilot study	10	AR, Magic Leap (Magic Leap Inc., FL, USA)	DIEP	Usability of AR according to validated SUS questionnaire	17/39
Necker et al.^39^	Pilot study	15	AR, HoloLens 1, (Microsoft, Redmond, WA)	DIEP	Intraoperative verification of perforators using AR versus handheld Doppler US using a 3D print as ground truth	11/39
Meier et al.^40^	Pilot study	50	AR projection, in-house created device	DIEP	Perforator identification with infrared thermography projective AR confirmed with handheld Doppler US, CTA and intraoperative verification	18/39

a RCT, Randomized controlled trial.

### XR modalities and reconstructed anatomy

**Figure 2:**
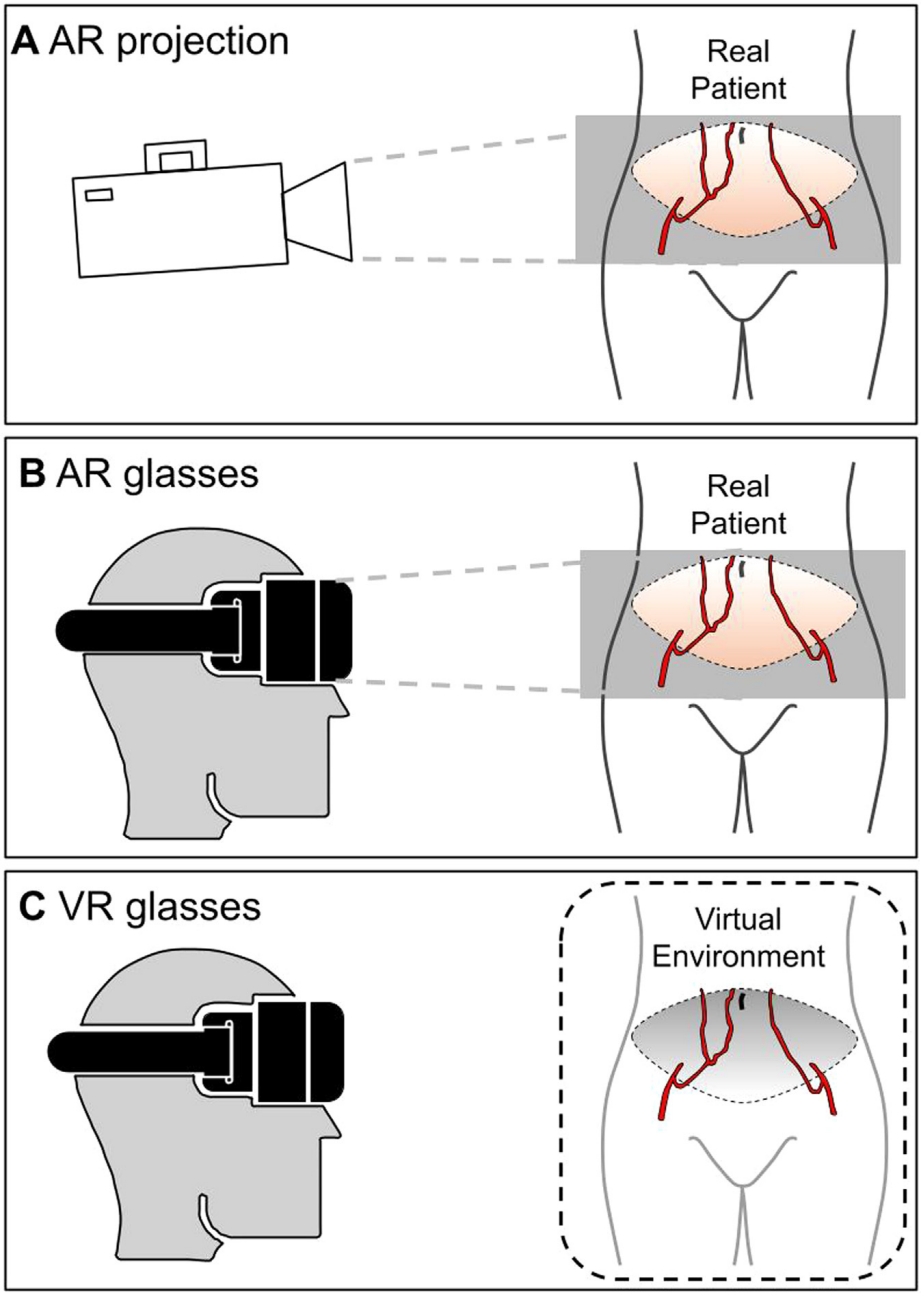
Extended reality modalities. A: Augmented reality projection. B: Augmented reality glasses. C: Virtual reality glasses.

Three distinct XR modalities were identified within the ten included articles: projection-based AR, AR glasses, and VR. Figure 2 provides an illustrative summary of different XR modalities used for ABR.

Projection-based AR, assessed in four articles, overlays virtual elements onto real-world physical surfaces without requiring a head-mounted device for interaction.^31^, ^32^, ^33^^,^^40^ These four studies were conducted at the same Department of Plastic Surgery, Radboud University Medical Centre (Nijmegen, the Netherlands) with the risk of overlapping study populations. However, each study included distinct, non-overlapping patient populations within these four articles.

AR glasses, described in five articles, use a headset to stereoscopically visualize virtual models within the user's real-world view.^34^^,^^35^^,^^37^, ^38^, ^39^

VR completely immerses users in a simulated environment through a headset, without registration onto the real world and was evaluated in one article.^36^

Eight articles utilized CTA images to reconstruct 3D models.^31^, ^32^, ^33^, ^34^, ^35^, ^36^, ^37^^,^^39^ The anatomical structures most often visualized in XR were the deep inferior epigastric artery (DIEA), the perforators, the umbilicus, the rectus abdominis muscle, and the intramuscular course of the perforators. Some articles also included structures such as the pelvis, xiphoid process, skin, and the superficial inferior epigastric artery (SIEA). Table 2 provides an overview of the XR modalities and the reconstructed anatomy in the virtual environment.

**Table 2:** XR modalities and reconstructed anatomy.

Study	XR modality	Segmentation software	Imaging modality for creation of the virtual model	Registration method	Reconstructed anatomy
Hummelink et al.^31^	AR projection	Vitrea software (Vital Images, Toshiba Medical Systems, Europe)	CTA	Handheld manual alignment using the umbilicus as reference	DIEA, perforators, branching pattern, umbilicus
Hummelink et al.^32^	AR projection	Vitrea software (Vital Images, Toshiba Medical Systems, Europe)	CTA	Automatic alignment using multiple markers	DIEA, perforators, branching pattern, skin
Hummelink et al.^33^	AR projection	Vitrea software (Vital Images; Toshiba Medical Systems Group Company, Minnetonka, Minn.)	CTA	Automatic alignment using multiple markers	DIEA, perforators, branching pattern, intramuscular course
Fitoussi et al.^34^	AR glasses	N/A^a^	CTA	Automatic alignment using a marker	DIEA, perforators, rectus abdominis muscle
Masterton et al.^35^	AR, glasses	TK-SNAP 3.8.0 software package	CTA	Manual registration using the umbilicus as reference point	Abdominal arteries, rectus abdominis muscle, perforators, skin
Freidin et al.^36^	VR	D2P software (3D Systems Inc., Littleton Colo.)	CTA	N/A^a^	SIEA. DIEA, intramuscular course, External Iliac artery, umbilicus, skin
Seth et al., ^37^	AR glasses	Materialize Mimics Innovation Suite (Materialise NV, Belgium)	CTA	Manual alignment using anatomical landmarks	SIEA, DIEA, perforators, intramuscular course, rectus abdominis muscle, pelvis, xiphoid process, skin
Berger et al.^38^, 2023	AR glasses	N/A^a^	MR-A	Manual alignment using anatomical landmarks	2D overlay, no 3D reconstruction
Necker et al.^39^	AR glasses	DICOM to PRINT-3D Systems (Rock Hill, S.C.)	CTA	Manual alignment using anatomical landmarks	DIEA, perforators, rectus abdominis muscle, skin
Meier et al.^40^	AR projection	N/A^a^	N/A^a^	Automatic alignment using multiple markers	N/A^a^

a N/A, not available/not applicable.

Berger et al. used MR-A as the imaging modality for the XR application.^38^ In contrast to the articles that employed CTA imaging to create 3D models, no models were constructed from the MR-A data. Instead, MR-A images were directly projected onto the patient in real-time using AR glasses.

Meier et al. employed infrared thermography to identify hotspots on the abdomen's skin, which were hypothesized to be correlated to perforator locations.^40^ These hotspots were projected real-time onto the patient's abdomen using an in-house created projection device. This approach distinguishes itself from other studies in this review, which used CTA or MR-A imaging to identify perforators.

### Perioperative perforator identification

Perioperative perforator identification rate was assessed in eight studies within this review.^31^, ^32^, ^33^^,^^35^^,^^37^, ^38^, ^39^, ^40^ The varying methods can be categorized into four groups.

The first method, intraoperative verification, was employed in four articles.^31^^,^^33^^,^^35^^,^^38^ Perforators were visualized using XR before surgery, and their location was confirmed intraoperatively. This experimental setup was used by Hummelink et al. (2015), Hummelink et al. (2019), Masterton et al., and Berger et al., and found perioperative perforator identification rates of 84.3 %, 61.7 %, 100 % and 100 % respectively.^31^^,^^33^^,^^35^^,^^38^ AR demonstrated significantly better perforator identification rates compared to handheld Doppler US in two studies by Hummelink et al.^31^^,^^33^

The second method, handheld Doppler US verification, was described in five articles^32^^,^^34^^,^^35^^,^^37^^,^^38^ Here, XR was used to visualize perforators, followed by handheld Doppler US to verify an arterial signal before surgery. Hummelink et al. (2017), Fitoussi et al., Masterton et al., Seth et al., and Berger et al. reported perforator identification rates using XR and verified by handheld Doppler US in 98 %, 92 %, 100 %, 80 %, and 100 % of the cases, respectively.^29^^,^^31^^,^^32^^,^^34^^,^^35^

The third method, described by Necker et al., evaluated the accuracy of perforator localization using AR glasses and a 3D-printed model of the patient's anatomy based on the CTA scan.^39^ The perforator points identified by AR were compared to those located by handheld Doppler US and referenced against the 3D print.^39^ AR demonstrated significantly higher accuracies, with an average deviation of 4.02 ± 3.20 mm compared to 9.71 ± 6.16 mm for handheld Doppler US.

The study by Meier et al. stands out due to its unique methodology.^40^ Researchers used infrared thermography to detect hotspots on patients' abdomens preoperatively. These hotspots were then marked and hypothesized to correspond to perforator locations. They identified a total of 514 hotspots amongst 50 patients. Of these 514 hotspots, arterial Doppler US signals were audible in 500 cases (97.3 %). Furthermore, 426 perforators were identified on CTA, of which 244 matched the locations of hotspots. Intraoperatively, 132 perforators were identified, of which 75 matched within a 2 cm range of a hotspot.^40^ All findings regarding the identification of perforator vessels can be found in Table 3.

**Table 3:** Perioperative perforator identification rate.

Study	Number of flaps	XR modality	Outcome measure	Perforator identification rate
Hummelink et al.^31^	N/A^a^	AR projection	Intraoperative verification of perforators using AR versus handheld Doppler US	AR: 84.3 % ± 25.8 % Doppler: 56.9 % ± 31.4 % (*p* = 0.030)
Hummelink et al.^32^	9	AR projection	Perforators identified with AR confirmed with handheld Doppler US	41/42 (98 %) perforators identified with AR were audible with Doppler
Hummelink et al.^33^	89	AR projection	Intraoperative verification of perforators using AR versus handheld Doppler US	AR: 61.7 % ± 7.3 % Doppler: 41.2 % ± 8.2 % (*p* = 0.020)
Fitoussi et al.^34^	N/A^a^	AR glasses	Perforators identified with AR confirmed with handheld Doppler US within 10 mm	24/26 (92 %) of perforators identified with AR were within 10 mm of the Doppler location
Masterton et al.^35^	2	AR glasses	Intraoperative verification of perforators using AR and handheld Doppler US	2/2 cases
Seth et al., ^37^	5	AR glasses	Perforators identified with AR confirmed with handheld Doppler US	4/5 cases
Berger et al.^38^	N/A^a^	AR glasses	Intraoperative verification of perforators using AR and handheld Doppler US	10/10 cases
Necker et al.^39^	N/A^a^	AR glasses	Distance of perforator markings using AR versus handheld Doppler US using a 3D print of the patient as ground truth	AR: 4.02 ± 3.20 mm Doppler: 9.71 ± 6.16 mm (*p* < 0.001)
Meier et al.^40^	71	AR projection	Perforator identification with infrared thermography projection AR confirmed with handheld Doppler US, CTA and intraoperative verification	500/514 (97.3 %) of the hotspots were audible with Doppler. 244/514 (47.5 %) were matched with CTA, and 75/514 (14.6 %) were perforators identified intraoperatively.

a N/A, not available/not applicable.

### Model reconstruction, planning, and operative time

Six studies examined the duration of 3D model reconstruction, pre-, peri‑, and/or intraoperative time.^33^^,^^35^, ^36^, ^37^, ^38^, ^39^ Hummelink et al. (2019) reported a significant reduction in intraoperative perforator localization times with AR (2.3 ± 0.8 min) compared to handheld Doppler US (20.0 ± 5.5 min) (*p* = 0.000). Additionally, they observed a significant decrease of 19 min in flap dissection times with AR (136 ± 7 min) compared to handheld Doppler US (155 ± 7 min) (*p* = 0.012).^33^

Two articles measured the duration of the preoperative registration or alignment process for the virtual 3D overlay. Seth et al. reported a time of 7–10 min, while Berger et al. found that patient detection and visualization took approximately 11 min.^37,^^38^

Preoperative 3D model construction took 15 min according to Masterton et al., 60 min as reported by Seth et al., and 45–60 min according to Freidin et al.^35^, ^36^, ^37^

Freidin et al. reported a preoperative planning duration for VR of 10 min.^36^ A complete overview of the planning and procedures duration can be found in Table 4.

**Table 4:** Model reconstruction, planning, and operative time.

Study	Number of flaps	XR modality	Outcome measure	Time in minutes
Hummelink et al.^33^	89	AR projection	Intraoperative mean perforator localization time: Intraoperative mean duration of flap harvest:	AR: 2.3 ± 0.8 Doppler: 20.0 ± 5.5 (*p* = 0.000) AR: 136 ± 7 Doppler: 155 ± 7 (*p* = 0.012)
Masterton et al.^35^	2	AR glasses	Preoperative reconstruction of 3D model duration:	15
Freidin et al.^36^	42	VR	Preoperative examination of virtual 3D model by surgeons: Preoperative reconstruction of 3D model duration:	10 45–60
Seth et al., ^37^	5	AR glasses	Preoperative time from CTA to AR reconstructed image: Preoperative manual alignment of virtual overlay: Preoperative drawing of the perforators with XR:	60 7–10 5–7
Berger et al.^38^	N/A^a^	AR glasses	Preoperative image preparation: Preoperative patient detection and visualization:	72 11
Necker et al.^39^	N/A^a^	AR glasses	Intraoperative mean perforator localization time:	AR: 1.28 ± 0.40 Doppler: 2.51 ± 0.51 (*p* < 0.001)

a N/A, not available/not applicable.

### Usability

Three articles assessed the usability of XR as experienced by surgeons.^35^^,^^36^^,^^38^ Masterton et al. descriptively reported the surgeons' experiences, using terms like ``convenient and uncomplicated''.^35^ Freidin et al. employed a self-designed questionnaire, with key outcomes showing that 72.4 % of surgeons rated VR models as having the highest similarity to reality, and 86.2 % expressed a strong interest in using VR for future cases.^36^

Berger et al. conducted the validated System Usability Scale (SUS) questionnaire, yielding a score of 67 out of 100 (SD: 10), indicating "moderate to good" usability.^38^ All findings regarding the usability are summarized in Table 5.

**Table 5:** Usability.

Study	Number of surgeons	XR modality	Outcome measure	Usability
Masterton et al.^35^	N/A^a^	AR glasses	Subjective and descriptive feasibility	Convenient, uncomplicated, simple-to-use
Freidin et al.^36^	3	VR	Usability of VR assessed through a self-developed questionnaire	In 72.4 % of cases VR models were rated as having the highest similarity to reality. In 86.2 % of cases surgeons expressed strong interest in using VR for future cases
Berger et al.^38^	two surgeons, one resident	AR glasses	SUS^b^	67, SD^c^: 10 ‘Moderate to good’

a N/A, not available/not applicable.

b SUS, System Usability Scale.

c SD, standard deviation.

### Complications

Four articles reported intra- and/or postoperative complications following the use of XR for ABR.^31^^,^^33^^,^^34^^,^^36^ No intraoperative complications were observed in two studies by Hummelink et al.^31^^,^^33^ In the RCT published by Hummelink et al. (2019), no significant difference was found between the prevalence of complications using handheld Doppler US or AR.^33^ Fitoussi et al. found no flap loss or partial necrosis in their cohort of 12 patients.^34^ Freidin et al. categorized complications into major and minor complications and found a prevalence of 1 % and 6 %, respectively.^36^ Comprehensive findings on complications are detailed in Table 6.

**Table 6:** Complications.

Study	Number of flaps	XR modality	Complication type	Prevalence:
Hummelink et al.^31^	9	AR projection	Intraoperative:	0
Hummelink et al.^33^	AR: 48 Doppler: 41	AR projection	Intraoperative: Flap loss: Flap revisions: Infection: Abdominal dehiscence:	AR: 0; Doppler: 0 AR: 2; Doppler: 1 AR: 3; Doppler: 2 AR: 2; Doppler: 1 AR: 6; Doppler: 7
Fitoussi et al. ^34^	N/A^a^	AR glasses	Flap loss, partial necrosis	0
Freidin et al.^36^	42	VR	Major complications: (Flap loss) Minor complications: (partial flap loss, infection, congestion)	1 6

a N/A, not available/not applicable.

### Costs

Four articles discussed costs related to XR.^33^^,^^35^^,^^37^^,^^39^ Hardware costs for XR glasses ranged from US $3500 to US $4141.94.^35^^,^^37^^,^^39^ Hummelink et al. conducted a cost-effectiveness analysis using a financial model, revealing a potential reduction of €298 per flap with AR projection, attributed to decreased operating time.^33^ Cost details are outlined in Table 7.

**Table 7:** Costs.

Study	XR modality	Outcome measure	Price
Hummelink et al.^33^	AR projection	Cost-reduction according to financial model	€298 saved per harvested flap^a^
Masterton et al.^35^	AR glasses	Costs of HoloLens 1 (Microsoft, Redmond, WA)	UK £3349 (US $4141.94)
Seth et al. ^37^	AR glasses	Costs of HoloLens 2 (Microsoft, Redmond, WA)	US $3500
Necker et al.^39^	AR glasses	Costs of HoloLens 2 (Microsoft, Redmond, WA)	US $3500

a Based on an in-article provided cost-effectiveness model.

## Discussion

This systematic review aimed to provide a comprehensive overview of the use of XR for perioperative perforator visualization in DIEP BR. This systematic review found that few studies have reported on the clinical application of XR in DIEP BR reconstruction, with varying quality. Different XR modalities are used, generally aiding in more accurate and faster perforator localization and compared to handheld Doppler US. Although XR models typically require more preoperative preparation time compared to CTA alone, surgeons involved in the included studies often reported a preference for XR due to its ability to provide a more intuitive and immersive understanding of patient-specific anatomy. However, it is important to acknowledge that these findings may be influenced by user selection bias, as the surgeons participating in these early-phase studies may have been more receptive to or enthusiastic about adopting new technologies.

In contrast to previous reviews by Lacey et al. and Ozmen et al., studies using 3D models viewed on standard 2D monitors were excluded, as these do not provide stereoscopic or immersive visualization.^26^, ^27^, ^28^ A stricter definition of XR was applied in this review, focusing on immersive technologies that offer depth perception. This distinction is considered essential, as stereoscopic XR allows for a more intuitive spatial understanding of patient-specific anatomy.^28^

Reported complication rates in the included studies of this systematic review do not suggest a negative impact of XR on surgical outcomes.^31^^,^^33^^,^^34^^,^^36^ In the four studies that addressed complications, no intraoperative complications were reported, and the overall rate of post-operative complications was comparable to complication rates observed in literature.^31^^,^^33^^,^^34^^,^^36^^,^^41^, ^42^, ^43^ Notably, in the only randomized controlled trial by Hummelink et al. (2019), complication rates did not significantly differ between XR and handheld Doppler US.^33^ Based on the available data, it appears unlikely that the use of XR was associated with an increased risk of complications. Of importance, data about postoperative complication has a marginal level of evidence due to the small sample sizes and methodology of the included pilot studies.

While perforator identification rate and perforator identification time using XR was found to be superior to handheld Doppler US, it is important to note that this comparison warrants careful interpretation, as CTA is the gold standard for perforator identification.^17^^,^^33^^,^^39^ Although CTA or MR-A data were available in the included studies, the authors of the included articles chose to compare XR primarily to handheld Doppler rather than to CTA itself. As such, it is unclear whether XR provides meaningful additional information beyond plotting CTA coordinates onto the patient’s abdomen which is fast, cost-effective, and widely used.^15^, ^16^, ^17^, ^18^

XR provides stereoscopic visualization and enhanced spatial understanding, however, the added depth perception may not translate into additional clinically relevant information, as the perforator transit through the rectus sheath, vessel caliber, and intramuscular course can be assessed effectively using conventional CTA.^15^, ^16^, ^17^, ^18^^,^^33^^,^^39^ Therefore, the incremental value of XR in routine preoperative mapping remains uncertain.

Moreover, several studies report perforator identification rates of up to 100 % when using XR.^35^^,^^36^ While promising, such results are likely influenced by reporting limitations or methodological bias, as perfect accuracy is improbable in clinical practice. These findings may reflect either selective reporting, subjective confirmation methods, or small sample sizes rather than true diagnostic precision.

No articles included in this systematic review directly compared total operative time between XR and CTA, the gold standard, alone. However, the duration of intraoperative perforator localization and flap dissection was significantly shorter with XR compared to handheld Doppler US.^33^ This reduction in time could potentially lower complication rates and costs associated with prolonged operative durations, as concluded in systematic reviews on the perioperative use of XR in other surgical specialties.^22^^,^^25^^,^^44^^,^^45^

XR hardware, when spread over multiple patients and indications, constitutes to only a minor fraction of the total costs since AR hardware costs between US $3500 and $4195 and is a one-time expense, that could be spread out over multiple years and used for varying purposes.^35^^,^^37^^,^^39^^,^^46^ However, it is important to acknowledge that hardware costs alone do not capture the full economic impact of XR integration. The cost-effectiveness of XR must also account for the need for image segmentation, 3D reconstruction, and system setup, all of which require time, software infrastructure, and trained personnel. A comprehensive cost-benefit analysis that includes hardware, software, imaging, processing time, and clinical outcomes is therefore necessary to determine the true economic feasibility of XR in routine surgical practice.

All included articles focused on DIEP flap ABR, even though the objective was to include all autologous free flap breast reconstructions utilizing XR. This highlights the prominence of DIEP flap ABR as the gold standard in breast reconstruction.^11^^,^^12^ Nevertheless, not all patients have adequate surplus tissue at the abdominal wall to qualify for DIEP flap BR.^6^, ^7^, ^8^, ^9^, ^10^, ^11^ In 2018, Pratt et al. extended the use of AR HoloLens beyond breast reconstruction to extremity defect flap reconstruction. Their findings demonstrated AR's ability to accurately localize perforators in regions other than the abdominal wall, emphasizing the potential of XR technology for patients ineligible for abdominal-based free flap BR.^47^

Finally, most articles included in this systematic review solely focus on preoperative perforator identification in ABR using XR. Wesselius et al. introduced a workflow for intraoperative AR navigation.^48^ However, the effectiveness of intraoperative navigation using AR for ABR is questionable without accounting for soft-tissue deformation, as demonstrated in other surgical fields such as spinal and oncological surgery.^49^^,^^50^ It may be possible that other factors such as muscle relaxation under anesthesia, and changes in body position relative to the CTA, also affect the accuracy of intraoperative AR navigation for flap harvest. Despite these obstacles, the development of non-rigid registration tools like Elastix shows promise in addressing these issues and improving the feasibility of intraoperative AR navigation in ABR.^51^

### Limitations

Firstly, a major limitation is the overall low level of evidence among the included studies. Nine out of the ten articles are pilot studies, which inherently carry a higher risk of bias and provide limited generalizability. Importantly, many of these early-phase studies were potentially conducted by technologically inclined teams with a strong interest in XR, which may introduce performance bias due to higher levels of expertise or enthusiasm. As such, these results may not accurately reflect outcomes in routine clinical practice.

Secondly, no standardized quality assessment exists for pilot study designs. The QuADS tool enables the evaluation of articles with heterogeneous study designs but is not specifically tailored for feasibility or pilot studies.^30^ As a result, even if a pilot study’s methodology is well described, its quality may still be rated relatively low due to the criteria used in the QuADS tool.

Additionally, the heterogeneity in experimental designs and outcome measures among the studies prevented the execution of a meta-analysis. Significant challenges were posed by this diversity in synthesizing the data for a unified and comparative analysis. Consequently, an effort was made to present the findings in a more structured manner by section, aiming for the highest degree of comparability possible under these constraints.

### Future research

Current evidence does not demonstrate clear clinical advantages of XR in DIEP BR over conventional CTA-based planning. Therefore, future research should conduct studies with higher methodological quality and stronger levels of evidence that compare XR, alone or combined with CTA, against CTA alone. This is crucial for objectively determining XR's potential advantages in enhancing perforator identification rate, reducing planning and operative times and lowering complication rates of free flap ABR. Furthermore, there is a need for standardized outcome measures and a uniform conceptual clarification of the term ‘XR’ to enable consistent evaluation of XR's clinical and economic impact. These steps are crucial for determining the true value of XR and guiding its integration into routine surgical practice.

## Conclusion

This systematic review suggests that XR is technically feasible for perioperative perforator visualization in DIEP flap breast reconstruction and may enhance anatomical understanding. However, current evidence is limited by small, low-quality studies that primarily compare XR to handheld Doppler rather than the gold standard CTA. It remains unclear whether XR provides clinically meaningful advantages over conventional CTA imaging.

## Ethics statement

Not required.

## Funding

This study did not receive any specific grant from funding agencies in the public, commercial, or not-for-profit sectors.

## Declaration of competing interest

None.

## Appendix Supplementary materials

Image, application 1

Image, application 2
