# Corrigendum to ‘Extended reality for perforator visualization in deep inferior epigastric perforator autologous breast reconstruction: A systematic review’ [JPRAS Open 48C (2026) 253–268]

**DOI:** 10.1016/j.jpra.2026.01.032

**Published:** 2026-01-27

**Authors:** Killian Zijlstra, H. Chien Nguyen, Koen Willemsen, J. Henk Coert, Eveline M.L. Corten, Wiesje Maarse

**Affiliations:** a Division of Surgical Specialties, University Medical Center Utrecht, 3D Lab, Utrecht, the Netherlands; b Department of Plastic, Reconstructive and Hand Surgery, University Medical Center Utrecht, Utrecht, the Netherlands; c Department of Orthopaedics, University Medical Center Utrecht, Utrecht, the Netherlands; d Department of Plastic, Reconstructive and Hand Surgery, Erasmus MC Cancer Institute, University Medical Center Rotterdam, Rotterdam, the Netherlands

The authors regret that the author names were incorrectly formatted in the published version of this article, resulting in incomplete representation of some initials. The correct author names are presented above, with the full first names retained and the missing initials included. The authors would like to apologize for any inconvenience caused.

