# Supplementary material for: Extended reality for perforator visualization in deep inferior epigastric perforator autologous breast reconstruction: A systematic review
Source: JPRAS Open. 2025 Nov 24;48:253–68. doi: 10.1016/j.jpra.2025.11.025 (PMC12752762; doi:10.1016/j.jpra.2025.11.025)
Supplement: Supplementary file 1 [file mmc1.docx]

# Appendix 1

Full search string

| **Database searched** | **Platform** | **Years of coverage** | **Records** | **Records after duplicates removed** |
| --- | --- | --- | --- | --- |
| Medline ALL | Ovid | 1946 - Present | 298 | 296 |
| Embase | Embase.com | 1971 - Present | 523 | 281 |
| Web of Science Core Collection* | Web of Knowledge | 1975 - Present | 215 | 21 |
| Cochrane Central Register of Controlled Trials | Wiley | 1992 - Present | 10 | 3 |
| Additional Search Engines: Google Scholar** | | | 100 | 55 |
| **Total** | | | **1146** | **656** |

*Science Citation Index Expanded (1975-present) ; Social Sciences Citation Index (1975-present) ; Arts & Humanities Citation Index (1975-present) ; Conference Proceedings Citation Index- Science (1990-present) ; Conference Proceedings Citation Index- Social Science & Humanities (1990-present) ; Emerging Sources Citation Index (2005-present)
**Google Scholar was searched via "Publish or Perish" to download the results in EndNote.

No other database limits were used than those specified in the search strategies

**Medline**

(exp "Augmented Reality"/ OR exp "Imaging, Three-Dimensional"/ OR exp "Virtual Reality"/ OR (3D OR 3-D OR three-dimension* OR threedimension* OR 3-dimension* OR 3dimension* OR ((augment* OR virtual* OR mixed* OR extend*) ADJ3 (realit*)) OR smart*-glas*).ab,ti,kf.) AND (((exp "Transplantation, Autologous"/ OR (autolog* OR fat* OR flap*).ab,ti,kf.) AND (exp "Mammaplasty"/)) OR (((autolog* OR fat* OR flap*) ADJ6 (breast* OR mamma* OR chest*) ADJ6 (reconstruct* OR surger* OR surgi* OR augment* OR graft* OR esthetic* OR aesthetic*))).ab,ti,kf.)

**Embase**

('augmented reality system'/exp OR 'three-dimensional imaging'/exp OR 'augmented reality'/exp OR 'virtual reality'/exp OR 'mixed reality'/exp OR 'extended reality'/exp OR (3D OR 3-D OR three-dimension* OR threedimension* OR 3-dimension* OR 3dimension* OR ((augment* OR virtual* OR mixed* OR extend*) NEAR/3 (realit*)) OR smart*-glas*):ab,ti,kw) AND ('autologous breast reconstruction'/exp OR (('autotransplantation'/exp OR (autolog* OR fat* OR flap*):ab,ti,kw) AND ('breast surgery'/exp OR 'breast reconstruction'/exp)) OR (((autolog* OR fat* OR flap*) NEAR/6 (breast* OR mamma* OR chest*) NEAR/6 (reconstruct* OR surger* OR surgi* OR augment* OR graft* OR esthetic* OR aesthetic*))):ab,ti,kw)

**Web of Science**

TS=(((3D OR 3-D OR three-dimension* OR threedimension* OR 3-dimension* OR 3dimension* OR ((augment* OR virtual* OR mixed* OR extend*) NEAR/2 (realit*)) OR smart*-glas*)) AND ((((autolog*

OR fat* OR flap*) NEAR/5 (breast* OR mamma* OR chest*) NEAR/5 (reconstruct* OR surger* OR surgi* OR augment* OR graft* OR esthetic* OR aesthetic*)))))

**Cochrane CENTRAL**

((3D OR 3 NEXT/1 D OR three NEXT/1 dimension* OR threedimension* OR 3 NEXT/1 dimension* OR 3dimension* OR ((augment* OR virtual* OR mixed* OR extend*) NEAR/3 (realit*)) OR smart* NEXT/1 glas*):ab,ti,kw) AND ((((autolog* OR fat* OR flap*) NEAR/6 (breast* OR mamma* OR chest*) NEAR/6 (reconstruct* OR surger* OR surgi* OR augment* OR graft* OR esthetic* OR aesthetic*))):ab,ti,kw)

**Google Scholar**

3d|'3|three d|dimensional'|'augmented|virtual|mixed|extended reality' 'autologous|fat|flap breast|mamma reconstruction|surgery|graft|augmentation'
