# Supplementary material for: Extended reality for perforator visualization in deep inferior epigastric perforator autologous breast reconstruction: A systematic review
Source: JPRAS Open. 2025 Nov 24;48:253–68. doi: 10.1016/j.jpra.2025.11.025 (PMC12752762; doi:10.1016/j.jpra.2025.11.025)
Supplement: Supplementary file 2 [file mmc2.docx]

Appendix 2 - QuADS tool

| QuADS Criteria | **Item** | **1** | **2** | **3** | **4** | **5** | **6** | **7** | **8** | **9** | **10** | **11** | **12** | **13** | **Total** |
| --- | --- | --- | --- | --- | --- | --- | --- | --- | --- | --- | --- | --- | --- | --- | --- |
| **Author** | **Year** | |  |  |  |  |  |  |  |  |  |  |  |  |  |
| Hummelink et al. | 2015 | 1 | 1 | 1 | 1 | 0 | 0 | 1 | 1 | 0 | 1 | 0 | 0 | 1 | **8** |
| Hummelink et al. | 2017 | 1 | 1 | 2 | 1 | 0 | 0 | 1 | 1 | 0 | 1 | 1 | 0 | 1 | **10** |
| Hummelink et al. | 2019 | 3 | 3 | 3 | 3 | 1 | 1 | 2 | 3 | 3 | 2 | 2 | 1 | 2 | **29** |
| Fitoussi et al. | 2021 | 1 | 1 | 1 | 1 | 0 | 0 | 1 | 1 | 0 | 0 | 0 | 0 | 1 | **7** |
| Freidin et al. | 2023 | 2 | 2 | 3 | 2 | 0 | 2 | 2 | 3 | 0 | 1 | 2 | 0 | 1 | **20** |
| Berger et al. | 2023 | 1 | 3 | 3 | 1 | 0 | 3 | 1 | 1 | 0 | 0 | 2 | 1 | 1 | **17** |
| Seth et al. | 2023 | 2 | 2 | 3 | 1 | 0 | 0 | 0 | 0 | 0 | 0 | 0 | 0 | 0 | **8** |
| Masterton et al. | 2023 | 1 | 0 | 1 | 0 | 0 | 0 | 0 | 0 | 0 | 0 | 0 | 0 | 1 | **3** |
| Necker et al. | 2024 | 2 | 1 | 1 | 1 | 0 | 0 | 1 | 1 | 0 | 1 | 1 | 1 | 1 | **11** |
| Meier et al. | 2024 | 2 | 2 | 3 | 2 | 0 | 1 | 1 | 2 | 0 | 1 | 1 | 1 | 2 | **18** |
| Scale (0-3): 0 = minimum score, 3 = maximum score per item  Score: 0 = minimum total score, 39 = maximum total score | | | | | | | | | | | | | | | |
